# Hypothermic Preservation of Adipose-Derived Mesenchymal Stromal Cells as a Viable Solution for the Storage and Distribution of Cell Therapy Products

**DOI:** 10.3390/bioengineering9120805

**Published:** 2022-12-14

**Authors:** André Branco, Ana L. Tiago, Paula Laranjeira, Maria C. Carreira, João C. Milhano, Francisco dos Santos, Joaquim M. S. Cabral, Artur Paiva, Cláudia L. da Silva, Ana Fernandes-Platzgummer

**Affiliations:** 1 Department of Bioengineering and Institute for Bioengineering and Biosciences (iBB), Instituto Superior Técnico, Universidade de Lisboa, 1049-001 Lisbon, Portugal; 2 Associate Laboratory i4HB—Institute for Health and Bioeconomy at Instituto Superior Técnico, Universidade de Lisboa, 1049-001 Lisbon, Portugal; 3 Flow Cytometry Unit, Department of Clinical Pathology, Centro Hospitalar e Universitário de Coimbra, 3000-075 Coimbra, Portugal; 4 Coimbra Institute for Clinical and Biomedical Research (iCBR), Faculty of Medicine, Pólo das Ciências da Saúde, University of Coimbra, 3000-548 Coimbra, Portugal; 5 Center for Innovative Biomedicine and Biotechnology (CIBB), Pólo das Ciências da Saúde, University of Coimbra, 3000-548 Coimbra, Portugal; 6 Center for Neuroscience and Cell Biology (CNC), University of Coimbra, 3004–504 Coimbra, Portugal; 7 Hospital São Francisco Xavier, Centro Hospitalar de Lisboa Ocidental, 1449-005 Lisbon, Portugal; 8 StemLab, S.A., 3060-197 Cantanhede, Portugal; 9 Ciências Biomédicas Laboratoriais, ESTESC-Coimbra Health School, Instituto Politécnico de Coimbra, 3045-043 Coimbra, Portugal

**Keywords:** mesenchymal stromal cells, cell encapsulation, hypothermic temperatures, fetal bovine serum, human platelet lysate, xenogeneic-free, hematopoietic support assay

## Abstract

Cell and gene therapies (CGT) have reached new therapeutic targets but have noticeably high prices. Solutions to reduce production costs might be found in CGT storage and transportation since they typically involve cryopreservation, which is a heavily burdened process. Encapsulation at hypothermic temperatures (e.g., 2–8 °C) could be a feasible alternative. Adipose tissue-derived mesenchymal stromal cells (MSC(AT)) expanded using fetal bovine serum (FBS)- (MSC-FBS) or human platelet lysate (HPL)-supplemented mediums (MSC-HPL) were encapsulated in alginate beads for 30 min, 5 days, and 12 days. After bead release, cell recovery and viability were determined to assess encapsulation performance. MSC identity was verified by flow cytometry, and a set of assays was performed to evaluate functionality. MSC(AT) were able to survive encapsulated for a standard transportation period of 5 days, with recovery values of 56 ± 5% for MSC-FBS and 77 ± 6% for MSC-HPL (which is a negligible drop compared to earlier timepoints). Importantly, MSC function did not suffer from encapsulation, with recovered cells showing robust differentiation potential, expression of immunomodulatory molecules, and hematopoietic support capacity. MSC(AT) encapsulation was proven possible for a remarkable 12 day period. There is currently no solution to completely replace cryopreservation in CGT logistics and supply chain, although encapsulation has shown potential to act as a serious competitor.

## 1. Introduction

Cell and gene therapies (CGT) have seen a significant growth in the past decades due to their unmatched potential to improve the treatment landscape of a large variety of diseases [1]. CGT products differ from traditional biopharmaceuticals since they are capable of a much more complex response to disease than small molecules or antibodies. CGT can restore tissues or increase the body’s innate ability to fight disease by dynamically reacting to environmental and biological cues [2].

Among potential CGT products, mesenchymal stromal cells (MSC) became the subject of great research interest, spurred mainly by their potential for application in regenerative medicine. Their differentiation potential into different lineages, significant in vitro expansion capacity, accessible isolation from multiple sources with few associated ethical issues (e.g., umbilical cord and adipose tissue [AT]), and their good safety and efficacy profiles from a variety of pre-clinical studies encouraged their increased use in human clinical trials, particularly between 2004 and 2011 [3]. However, despite the large number of clinical trials, and contrary to expectations, the lack of statistically significant results in terms of efficacy and their discrepancy with the results of pre-clinical assays hampered the advancement of MSC-based therapies as marketed CGT [3,4]. It was only in 2018 that the European Medicines Agency (EMA) approved Alofisel, which is an allogenic MSC-based therapy for complex perianal fistulae in Crohn’s disease [5].

The success of Alofisel contrasted with the underwhelming results of previous human clinical trials and could be explained by a paradigm shift in the mechanism of the action of MSC. Focus changed from their tissue regeneration potential to their immunomodulatory action which leveraged their complex secretome and opened the door for the first statistically significant results in human clinical trials using MSC [6,7]. Due to this, and despite their initial disappointing results, MSC are again being used in a great number of clinical studies, and a great majority of them are in phase I and II (a search in clinicaltrials.gov in July 2022 for the term “Mesenchymal” yielded 1720 studies). The high number of ongoing early phase clinical trials suggests there is great untapped potential for more MSC-based CGT to be developed and approved.

Considering the vast therapeutic potential of MSC, from graft vs. host disease (GvHD) to spinal cord injury, the establishment of a robust manufacturing process that is good manufacturing practices (GMP)-compliant, minimizes variability and facilitates the approval of new CGT should be a priority, ensuring a quick translation from bench-to-bedside [1,2,6,7,8]. Furthermore, CGT usually have higher costs compared with other classes of medicines; therefore, to ensure commercial effectiveness, it is important to consider cost of goods (COGs) optimization in the early stages of process development with the aim of minimizing the cost per dose without compromising the product quality. In the specific case of allogeneic MSC-based products, these medicines usually require large manufacturing scales, so opportunities for economies of scale can be leveraged [9,10,11]. Indeed, the large-scale production of allogeneic MSC is not a straightforward process, and the decisions regarding production platform design have a great impact in the robustness, validation, and commercial viability of the cell product.

Some critical steps in MSC production are their storage and journey from bench-to-bedside [12]. The processes used for storage and transportation must ensure the cell product is consistently GMP-compliant and safe to maintain cell viability and potency during the time window that separates the release of the product from a GMP facility to the clinical trial center or therapeutic facility [7,13,14].

Cryopreservation is currently regarded as an indispensable step of CGT production because it is a feasible strategy that allows for an MSC-based product to be an off-the-shelf product that can meet economical, logistical, and regulatory requirements. Cells can be stored in controlled conditions for extended periods and shipped in a frozen state to healthcare centers where they can be thawed and quickly administered to patients [14,15,16]. However, cryopreservation and thawing procedures are known to induce cellular injuries that negatively affect the stability and therapeutic efficacy of MSC-based products, which is possibly responsible, at least partially, for early disappointing clinical results [15,17].

Although cryopreservation is currently considered the gold standard in MSC manufacturing, finding alternative strategies that can replace and/or complement this process (serving as transportation solutions between production and healthcare providing facilities) has been the focus of several studies. Lyophilization, or freeze-drying, has been investigated as a possible strategy to store cells at an ambient temperature by rapidly freezing and dehydrating cells while using a protective compound, such as the sugar trehalose [18]. This method efficiently preserved placental tissue at room temperature (RT), which suggests that it can be a viable alternative for the preservation and storage of cellular products, including MSC, as it will simplify storage [19]. In cases where an extended shelf-life is not required, the hypothermic storage (between 2 and 8 °C) of cell products is perhaps a simpler solution as it relies only on storing and/or transporting cells in culture media designed specifically for this purpose. Multiple studies have shown the ability of research and clinical-grade media to maximize cell viability and function while storing them for periods between 1 and 7 days at temperatures around 4 °C [20,21,22]. Another popular strategy that relies on hypothermic storage for safely storing and transporting cells is encapsulation in different polymers, both natural and synthetic, with alginate being the most common [12].

Alginate is a natural polysaccharide that can be obtained from seaweed and jellified when cations are added to generate a biocompatible hydrogel [23]. The advantages of alginate as a simple and cost-effective solution for protecting cell products during storage and transportation have been attributed to its ability to stabilize the membrane of cells in suspension and to protect them from osmotic shock and mechanical stress [12,24]. Alginate encapsulation has potential to impact CGT manufacturing. Cells would be encapsulated in beads, stored at hypothermic temperatures in a non-proliferative stage, and then recovered by dissolving the gel and replacing the solutions with fresh culture media.

In this study, we aim to determine the ability of alginate encapsulation to maintain cell viability, identity, and function in the context of MSC-based therapy manufacturing. For this purpose, adipose tissue-derived MSC (MSC(AT)) were encapsulated and stored for a total of 12 days at hypothermic temperatures. MSC(AT) expanded in medium supplemented with human platelet lysate (HPL) were compared with cells expanded in medium supplemented with fetal bovine serum (FBS), which is the historical standard, to establish a xeno(geneic)-free condition in line with GMP requirements. To our knowledge, this is the first study to push MSC(AT) encapsulation to relevant time periods (i.e., 12 days) using a standardized, commercially available kit (BeadReady™, Atelerix, Newcastle, UK) that complies with GMP conditions. Encapsulated MSC(AT) were extensively analyzed to determine if their identity and function were preserved for the tested conditions in comparison to non-encapsulated cells.

## 2. Materials and Methods

### 2.1. Human Tissues

Adipose tissue (AT) samples and umbilical cord blood (UCB) units were obtained through collaboration agreements secured by the Institute for Bioengineering and Biosciences (iBB) at Instituto Superior Técnico (IST), Clínica de Todos-os-Santos (Lisbon), and Hospital São Francisco Xavier (Lisbon). Informed consent was obtained from healthy donors before the harvesting of samples in accordance with Directive 2004/23/EC of the European Parliament and of the Council of 31 March 2004 regarding standards of quality and safety for the donation, procurement, testing, processing, preservation, storage, and distribution of human tissues and cells, represented by the counterpart Portuguese Law 22/2007. Adipose tissue-derived mesenchymal stromal cells (MSC(AT)) isolation, characterization, and cryopreservation was performed following protocols previously established by the Stem Cell Engineering Research Group at iBB [25,26].

### 2.2. MSC(AT) Expansion

Cryopreserved MSC(AT) were thawed and seeded in low glucose Dulbecco’s Modified Eagle’s Medium (DMEM) (Thermo Fisher Scientific, Waltham, MA, USA) supplemented with 10% (*v*/*v*) MSC-qualified fetal bovine serum (FBS) (Thermo Fisher Scientific) and 1% (*v*/*v*) Antibiotic-Antimycotic (A/A) (Thermo Fisher Scientific) (DMEM-FBS) at a cell density of 3000 cells/cm^2^ on standard tissue culture plastic and were transferred to an incubator at 37 °C and 5% CO_2_ in a humidified atmosphere. The DMEM-FBS expansion medium was changed every 3–4 days until cells reached between 70 and 80% confluence. Cells were detached from their culture surface with 0.05% (*v*/*v*) trypsin (Thermo Fisher Scientific) and 1 mM ethylenediaminetetraacetic acid (EDTA) (Sigma-Aldrich, St. Louis, MI, USA) in phosphate buffered saline (PBS) (Sigma-Aldrich). In order to establish a xenogeneic-free culture condition, detached cells were evenly split between the existing DMEM-FBS condition and low glucose DMEM supplemented with 5% (*v*/*v*) human platelet lysate (HPL) (UltraGRO™-PURE, kindly provided by AventaCell Biomedical Corp., Atlanta, GO, USA) and 1% (*v*/*v*) A/A (DMEM-HPL). Adaptation to xeno-free conditions was completed after an additional DMEM-HPL passage for MSC(AT). Cell detachment under xeno-free conditions was performed using TrypLE (Thermo Fisher Scientific).

### 2.3. MSC(AT) Encapsulation

MSC(AT) encapsulation in alginate beads was performed following the instructions included in the BeadReady™ kit (kindly provided by Atelerix, Newcastle, UK). Briefly, the procedure was separated into three sections, including gelation, storage/transportation, and release. For each single encapsulation, 6 × 10^6^ cells were suspended in their respective medium at twice the final encapsulation density and then carefully mixed with an alginate solution. Slowly, the mixture was dropwise transferred into a gelation solution using a needle in order to form uniform and spherical beads. After gelation stabilization, beads were washed and stored in their respective expansion medium in a tightly sealed tube away from light at a temperature between 10 and 20 °C to mimic possible temperature oscillations during transportation. During encapsulation, medium samples were taken from each bead-containing tube, centrifuged, and stored at −80 °C for future metabolic analysis. Beads were dissociated 30 min after encapsulation (D0), at day 5 (D5), and at day 12 (D12). Cell release was done by replacing the expansion medium with the provided dissolution buffer. Afterward, released cells were washed and resuspended in fresh expansion medium. Recovered MSC(AT) were quantified to determine bead recovery, and their viability was also assessed.

### 2.4. Glucose and Lactate Profiles

Medium samples were thawed at room temperature (RT) and vortexed before they were distributed on a 96-well culture plate in duplicates. Glucose and lactate concentrations were determined using membrane-bound immobilized enzyme quantification in a YSI 2500 Biochemistry Analyzer (YSI, Yellow Springs. OH, USA). First-order regressions were fitted to MSC(AT) glucose and lactate concentration profiles. MSC(AT) glucose consumption and lactate production rates were determined from regression slopes multiplied by the expansion medium volume (5.5 mL) in bead-containing tubes. MSC(AT) specific glucose consumption and lactate production rates were calculated by dividing glucose and lactate rates by the cell number at each encapsulation timepoint. Glucose consumption and lactate production rates for MSC(AT)-hematopoietic stem cells and progenitor cells (HSPC) co-culture and HSPC culture control (No FL) were calculated by dividing the difference in concentration at the beginning and the end of the co-culture by the duration period of the hematopoietic support assay (7 days).

### 2.5. MSC(AT) Immunophenotype

Released and non-encapsulated MSC(AT) were sent in their respective expansion mediums at 4 °C to the Flow Cytometry Unit at Centro Hospitalar e Universitário de Coimbra where their immunophenotype was analyzed via flow cytometry on a FACSCanto II cytometer (BD Biosciences, Franklin Lakes, NJ, USA) using FACSDiva software (v8.02, BD Biosciences). For each condition, cells were resuspended in 100 μL PBS and stained for cell surface markers resorting to a stain–lyse–wash direct immunofluorescence technique. MSC(AT) were stained with the following antibodies: HLA-DR (L243) V450 (BD Biosciences), CD274 (29E.2A3) BV421 (BioLegend, San Diego, CA, USA), CD108 (KS-2) BV421 (BD Biosciences), CD45 (2D1) V500-C (BD Biosciences), CD73 (AD2) FITC (BioLegend), CD44 (L178) FITC (BD Biosciences), CD105 (TEA3/17.1.1) PE (Beckman Coulter, Brea, CA, USA), CD39 (TU66) PE (BD Biosciences), STRO-1 (STRO-1) PE (ExBio, Prague, Czech Republic), CD54 (LB-2) PE (BD Biosciences), CD34 (8G12) PerCP-Cy5.5 (BD Biosciences), CD200 (OX-104) PerCP-Cy5.5 (BioLegend), CD146 (SHM-57) PerCP-Cy5.5 (BioLegend), ICOSL (2D3) PerCP-Cy5.5 (BioLegend), CD19 (J3-119) PE-Cy7 (Beckman Coulter), CD271 (ME20.4) PE-Cy7 (BioLegend), CD106 (STA) PE-Cy7 (BioLegend), CD90 (5E10) APC (BioLegend), CD142 (NY2) APC (BioLegend), CD14 (MφP9) APC-H7 (BD Biosciences), B7-H4 (MIH43) APC Fire 750 (BioLegend), and CD10 (HI10α) APC-H7 (BD Biosciences). MSC(AT) were surface stained for 10 min in the dark and at RT. Following this step, cells were incubated for 10 min in the dark at RT with 2mL of FACSLysing solution (BD Biosciences) and then centrifuged, which discarded the supernatant. Cell pellets were then washed with PBS and resuspended so they were ready to be acquired. Following acquisition, data analysis was performed using Infinicyt (Cytognos, Salamanca, Spain), version 2.0.

### 2.6. MSC(AT) Multilineage Differentiation

#### 2.6.1. Adipogenic Differentiation

Released and non-encapsulated cells were plated at a density of 100,000 cells/cm^2^. After 24 h, differentiation was induced with the StemPro™ Adipogenesis Differentiation Kit (Thermo Fisher Scientific). Complete differentiation medium supplemented with 1% (*v*/*v*) A/A was changed twice a week for 21 days. Cells were then washed with PBS, fixed with 4% (*v*/*v*) paraformaldehyde (Sigma Aldrich) for 30 min at RT, and washed again with PBS. Fixed cells were initially incubated with a 60% isopropanol (Thermo Fisher Scientific) solution for 5 min at RT. Then, cells were stained to determine their degree of adipogenesis with a mixture (3:2) of 0.3% (*v*/*v*) Oil Red O (Sigma Aldrich) in a 60% (*v*/*v*) isopropanol solution and water for 1 h at RT. After incubation, cells were washed three times with distilled water and kept in PBS. Differentiation phenotype was observed under the microscope (DMI3000 B (Leica, Wetzlar, Germany)) and images were taken.

#### 2.6.2. Osteogenic Differentiation

MSC(AT) were seeded at a density of 100,000 cells/cm^2^ on a 24-well culture plate. Differentiation was also induced 24 h later by using the StemPro™ Osteogenic Differentiation Kit (Thermo Fisher Scientific). Complete differentiation medium supplemented with 1% (*v*/*v*) A/A was changed twice a week for 21 days, after which cells were fixed as stated for the adipogenic differentiation protocol. Cells were subjected to both an alkaline phosphatase and a Von Kossa stain to verify their osteogenic differentiation. Firstly, fixed cells were incubated for 40 min at RT in a solution of Fast Violet (Sigma Aldrich) and Naphthol (Sigma Aldrich), and then they were washed with distilled water afterward. With the alkaline phosphatase stain completed, cells were then incubated for 30 min with silver nitrate (Sigma Aldrich), washed three times with distilled water, and kept in PBS. Differentiation phenotype was observed under the microscope and images were taken.

#### 2.6.3. Chondrogenic Differentiation

For the chondrogenic differentiation, released and non-encapsulated MSC(AT) (800,000 cells in each condition) were prepared for aggregation using the hanging drop method. Cells were resuspended in 240 µL and droplets of 30 µL were placed on a Petri dish lid after being centrifuged and having their supernatant discarded. The droplet-containing lid was inverted onto its respective dish after filling the bottom of the Petri dish with PBS. Petri dishes were placed at 37 °C for 24 h for cell aggregates to form. Then, MSC(AT) aggregates were transferred onto Costar^®^ ultra-low attachment plates (Corning, Corning, NY, USA), and differentiation was induced with the MesenCult™-ACF Chondrogenic Differentiation Kit (STEMCELL Technologies, Vancouver, BC, Canada). Complete chondrogenic differentiation medium supplemented with 1% (*v*/*v*) A/A was changed twice a week for 21 days. Cells were incubated for 1 h in an 1% (*v*/*v*) Alcian Blue (Sigma Aldrich) solution, washed three times with distilled water, and kept in PBS to assess chondrogenic differentiation. Cells were observed under the microscope and images were taken.

### 2.7. Hematopoietic Support Assay

#### 2.7.1. UCB Mononuclear Cell (MNC(CB)) Isolation

MNC(CB) were isolated from UCB by phase separation using a Ficoll (GE Healthcare, Chicago, IL, USA) density gradient. After centrifugation, the layer of MNC(CB) was aspirated and washed with 2 mM EDTA (Sigma-Aldrich) in PBS. Removal of potential erythrocyte contamination was achieved by incubating cells at 4 °C with 155 mM ammonium chloride for 10 min. Isolated MNC(CB) were frozen in DMEM-FBS with 10% dimethyl sulfoxide (DMSO) and safely stored in a liquid/vapor phase nitrogen tank.

#### 2.7.2. Generation of a Cryopreserved CD34^+^ Pool from MNC(CB)

Six different donors of previously isolated MNC(CB) were thawed and pooled for CD34 enrichment through Magnetic Activated Cell Sorting (MACS) using the CD34 MicroBead Kit (Miltenyi Biotec, Bergisch Gladbach, Germany) according to the manufacturer’s instructions to generate a pool of CD34^+^ expressing cells for the entire study. CD34^+^ expression was confirmed by flow cytometry and a quality criterion of at least 70% CD34 expression was defined. Enriched cells were also subjected to a colony-forming unit (CFU) assay and a complete immunophenotypic analysis before being refrozen in DMEM-FBS supplemented with 10% DMSO and stored in a liquid/vapor phase nitrogen tank.

#### 2.7.3. MSC(AT) Feeder Layer Preparation

Released and non-encapsulated MSC(AT) were seeded at a density of 100,000 cells/cm^2^ on 12-well plates and left to adhere in an incubator overnight. The day after, confluent feeder layers were selected for the hematopoietic support assay.

#### 2.7.4. Ex Vivo Expansion of HSPC

For each expansion, a fraction of the pool of UCB-derived CD34^+^-enriched cells was thawed and seeded at a density of 30,000 cells/mL, both with the presence of an MSC(AT) feeder layer (co-culture) and without (no feeder layer control [No FL]). HSPC were expanded for 7 days in StemSpan Serum-Free Expansion Medium (SFEM) II (STEMCELL Technologies, Vancouver, BC, Canada) (2 mL/well) supplemented with 1% (*v*/*v*) A/A and a cytokine cocktail consisting of stem cell factor (SCF), fms-like tyrosine kinase 3 ligand (Flt-3L), thrombopoietin (TPO), and basic fibroblast growth factor (bFGF) (PeproTech, Cranbury, NJ, USA) with concentrations of 90, 77, 82, and 5 ng/mL, respectively. The cytokine concentrations that were used were previously optimized by our group targeting maximization of the expansion of UCB-derived CD34^+^-enriched cells in co-culture with MSC [27].

#### 2.7.5. Proliferation Assay

After 7 days, expanded HSPC (suspended and adhered) from each well were harvested through forced pipetting and counted to assess proliferation by Trypan Blue (Thermo Fisher Scientific) exclusion method. Fold change (FC) was calculated by dividing the number of expanded HSPC by the number of HSPC originally seeded (60,000 cells). Proliferation values were then normalized by dividing each FC by the one obtained for its respective No FL control.

#### 2.7.6. In Vitro Clonogenic Assay

HSPC potential to proliferate as colonies and differentiate into myeloid lineages was evaluated before and after HSPC expansion through the CFU assay. 1000 non-expanded CD34^+^-enriched cells (day 0) or 2500 expanded cells (day 7) were resuspended in 2 mL of MethoCult™ Classic (STEMCELL Technologies) medium, divided into three wells of a 24-well culture plate and left for 14 days to incubate at 37 °C, 5% CO_2_ in a humidified atmosphere [27]. After an incubation period of 14 days, multilineage colony-forming unit (CFU-Mix), burst-forming unit erythroid (BFU-E), and colony-forming unit granulocyte-macrophage (CFU-GM) colonies were classified and counted using a brightfield microscope (Olympus CK40 (Olympus, Tokyo, Japan)). Colony number was divided by the number of seeded cells and then multiplied by the number of expanded or non-expanded HSPC. Fold change in total colony number (FC Total CFU) was obtained by dividing the total colony number at day 7 by the respective of day 0.

#### 2.7.7. HSPC Immunophenotype

The immunophenotype of isolated and expanded HSPC was analyzed by flow cytometry. Briefly, cells were washed with PBS and viability was evaluated using a Far Red Fixable Dead Cell Stain Kit (Thermo Fisher Scientific). Afterward, cells were surface stained using previously titrated CD45RA (HI100) FITC (BD Biosciences), CD90 (5E10) PE (BioLegend) and CD34 (8G12) PerCP-Cy5.5 (BD Biosciences). Stained cells were acquired on a FACSCalibur™ cytometer (BD Biosciences). Data was analyzed using FlowJo v10 software (FlowJo LLC, Ashland, OR, USA).

### 2.8. Statistical Analysis

Data were analyzed using GraphPad Prism 8 software (Dotmatics, Boston, MA, USA). The results are presented as mean ± standard error of the mean (SEM). First-order regressions were calculated using the least squares regression fitting method. Goodness of fit was evaluated using the coefficient of determination (R^2^). A Shapiro–Wilk test was carried out to assess data normality for statistical hypothesis testing. One-way analysis of variance (ANOVA) was used to detect significant differences, and a Tukey multiple comparison test was done to determine which specific groups had statistical significance.

## 3. Results

**Figure 1:**
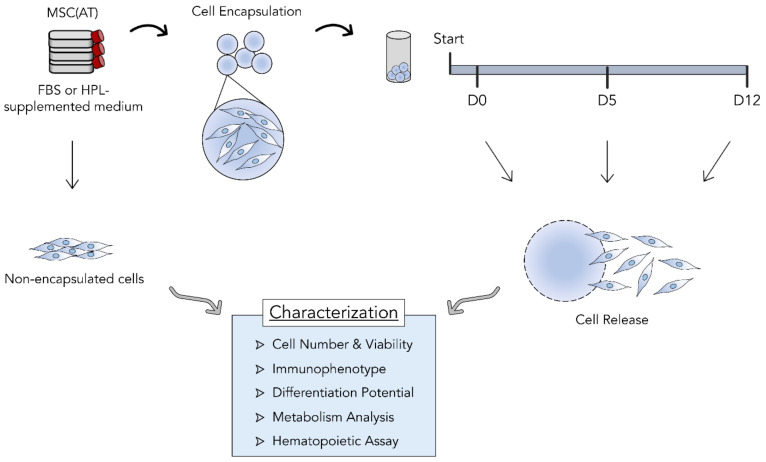
Study design. Three different adipose tissue-derived mesenchymal stromal cell (MSC(AT)) donors were expanded in fetal bovine serum (FBS) or human platelet lysate (HPL)-supplemented expansion medium in standard tissue culture plastic. MSC(AT) were encapsulated in alginate beads and kept at temperatures between 10 and 20 °C after reaching desired temperatures. MSC(AT) were left encapsulated during three different time periods: 30 min (D0), 5 days (D5), and 12 days (D12). Cells were then released and subjected to different characterization assays and compared with non-encapsulated MSC(AT). Cell retainment and survival during encapsulation, MSC identity and functional immunophenotype, MSC tri-lineage differentiation potential, metabolic activity, and hematopoietic support capacity were determined and compared between timepoints.

The feasibility of encapsulating mesenchymal stromal cells (MSC) as a means of storage and transportation for cell therapies was tested. Three independent donors of adipose tissue-derived MSC (MSC(AT) were expanded in vitro either in standard fetal bovine serum (FBS)-supplemented medium or xeno-free, human platelet lysate (HPL)-supplemented medium. For each condition, cells were encapsulated in alginate beads using the BeadReady™ kit. Encapsulated cells were maintained in an environment simulating transportation and storage conditions during three different time periods, including 30 min (D0), 5 days (D5), and 12 days (D12). Encapsulated cells were released and compared to non-encapsulated cells concerning their cell number, immunophenotype, metabolism, differentiation potential, and hematopoietic support capacity when they reached their specific timepoints (Figure 1).

### 3.1. MSC(AT) Were Successfully Encapsulated and Able to Withstand Hypothermic Temperatures for up to 12 Days

**Figure 2:**
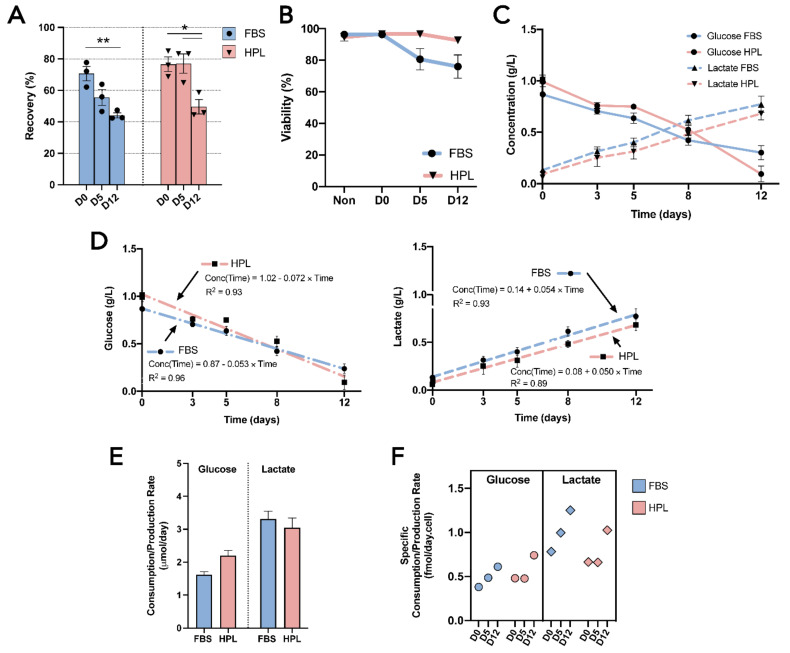
Cell encapsulation performance and MSC(AT) metabolic analysis. (**A**) Cell recovery from alginate beads after 30 min (D0), 5 days (D5), and 12 days (D12) for MSC-FBS (blue) and MSC-HPL (red). (**B**) Cell viability of MSC(AT) before encapsulation (Non) and after their release from encapsulation at D0, D5, and D12. (**C**) Glucose and lactate concentration profiles. (**D**) Glucose (**left**) and lactate (**right**) profile regression modelling, including the fitting of first-order regressions with the presentation of the equation and coefficient of determination (R^2^). (**E**) Molar glucose consumption and lactate production rates. (**F**) Specific molar glucose consumption and lactate production rates at the various encapsulation timepoints. (Three MSC(AT) donors; mean ± SEM; * *p* < 0.05, ** *p* < 0.01.)

A commercially available encapsulation kit, BeadReady™, which is based on alginate beads, was tested using adipose tissue-derived mesenchymal stromal cells (MSC(AT)) as target cells. An initial timepoint of 30 min of encapsulation (D0) was defined to quantify the encapsulation efficiency (i.e., the ratio of the encapsulated cells at D0 and the initial cell number prior to encapsulation). MSC(AT) expanded in fetal bovine serum (FBS)-supplemented medium (MSC-FBS) reached a 71 ± 5% efficiency, whereas MSC(AT) expanded in human platelet lysate (HPL)-supplemented medium (MSC-HPL) achieved a 77 ± 5% encapsulation efficiency (Figure 2A). After closely following the encapsulation protocol, formed beads were stable throughout the duration of the study, with no unwanted bead loss being observed. Cell recovery decreased as storage time increased for both MSC-FBS and MSC-HPL, reaching 44 ± 2% and 50 ± 5% at D12, respectively. Interestingly, MSC-FBS cell recovery showed a gradual drop throughout the timepoints (D0—71 ± 5% vs. D5—56 ± 5% [*p* > 0.082]; D5 vs. D12—44 ± 2% [*p* > 0.194]; and D0 vs. D12 [*p* < 0.01]), while MSC-HPL appeared to show a more stable encapsulation profile with no detectable cell loss within the first five days (D0—77 ± 5% vs. D5—77 ± 6%; D5 vs. D12—50 ± 5% [*p* < 0.05]; and D0 vs. D12 [*p* < 0.05]). Nevertheless, from D5 to D12, MSC-HPL showed a sharper decline as it reached similar levels of cell recovery at D12 as MSC-FBS.

Cell viability of encapsulated cells was also tracked throughout all timepoints (Figure 2B). Before encapsulation, a high viability was guaranteed (96 ± 0.3% for MSC-FBS and 95 ± 3% for MSC-HPL). Overall, the recovered cells maintained their viability during the 12 days, although MSC-FBS displayed a slight reduction trend to 76 ± 8% at D12, which somewhat mimicked the trend observed for cell recovery.

These results show that MSC(AT) can be encapsulated and survive in hypothermic temperatures, albeit with some cell loss occurring over time and a decrease in viability, particularly when working with MSC-FBS.

### 3.2. Encapsulated MSC(AT) Demonstrated an Active Metabolism Regardless of the Expansion Medium

During encapsulation, adipose tissue-derived mesenchymal stromal cells (MSC(AT)) showed a coherent consumption of glucose and a production of lactate between cell donors (Figure 2C). Neither biological variability nor expansion medium choice (MSC(AT) expanded in fetal bovine serum (FBS)-supplemented medium (MSC-FBS) vs. MSC(AT) expanded in human platelet lysate (HPL)-supplemented medium (MSC-HPL), which were causes of different MSC(AT) metabolic profiles. The consumption of glucose and the production of lactate displayed mirrored behavior, with steady decreases and increases as the encapsulation time grew. By D12, glucose levels were residual, thus exhibiting signs of nutrient exhaustion.

Metabolic profiles were subjected to regression analysis to better assess the metabolism of MSC(AT) while encapsulated. Both the glucose and lactate curves were successfully fit to individual linear functions with high correlation coefficients (R^2^ = [0.89–0.96]) (Figure 2D). Encapsulated MSC-FBS and MSC-HPL displayed constant glucose consumption and lactate production rates throughout the encapsulation as a consequence of possessing linear regression functions (Figure 2E). Interestingly, MSC-HPL consumed 2.2 ± 0.2 μmol glucose/day, which was higher than MSC-FBS with 1.6 ± 0.1 μmol glucose/day. In contrast, lactate production rates were very similar between MSC-FBS and MSC-HPL conditions.

Specific consumption and production rates were determined to better understand these metabolic rates at a cellular level (Figure 2F). Specific metabolic rates generally increased with encapsulation time since MSC(AT) had constant metabolic rates and cell recovery decreased over time. Specific glucose consumption rates for both MSC-FBS and MSC-HPL varied between 0.38 and 0.74 fmol/day.cell and specific lactate production rates between 0.65 and 1.25 fmol/day.cell. Apparent lactate/glucose yields (Y_lactate/glucose_) reflect previously mentioned differences in glucose consumption between MSC-FBS and MSC-HPL, with values of 2.05 and 1.38, respectively.

### 3.3. Upon Encapsulation, Released MSC(AT) Maintained Their Identity, Immunosuppressive Potential and Clonogenic Capabilities as Well as Their Differentiation Potential

**Figure 3:**
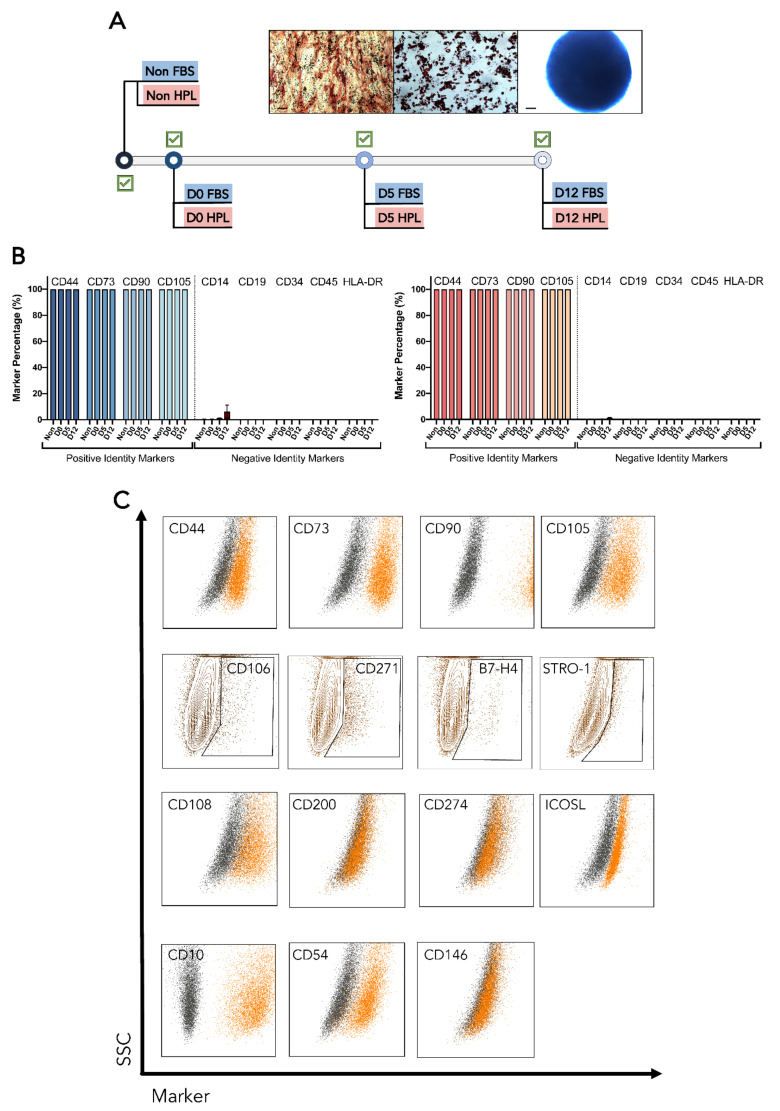
Differentiation potential and immunophenotype of MSC(AT) before and after encapsulation. (**A**) Map of MSC(AT) tri-lineage differentiations showing successful differentiation in every timepoint. The representative images include osteogenic (**left**), adipogenic (**center**), and chondrogenic (**right**) stainings. (**B**) Positive and negative identity marker expressions for MSC-FBS (**left**) and MSC-HPL (**right**) (%). (**C**) Representative MSC(AT) marker expressions for a defined encapsulation timepoint. Dotplots containing stained cells (orange) were overlaid with the unstained control (dark grey) for homogeneous populations with no subpopulations identified (first, third, and fourth row). Marker expressions that led to MSC(AT) positive subpopulations were gated in contour plots (second row). Scale bar: 100 μm; Non—non-encapsulated; SSC—Side scatter; MFI—median fluorescence intensity (three MSC(AT) donors; mean ± SEM).

After being encapsulated, recovered adipose tissue-derived mesenchymal stromal cells (MSC(AT)) preserved their ability to differentiate into osteogenic, chondrogenic, and adipogenic lineages. Following a 21-day differentiation, every biological donor from MSC(AT) expanded in fetal bovine serum (FBS)-supplemented medium (MSC-FBS) and MSC(AT) expanded in human platelet lysate (HPL)-supplemented medium (MSC-HPL) was able to successfully originate cells from each one of the three lineages (Figure 3A). Thus, no changes in MSC(AT) differentiation potential were observed after encapsulation.

**Figure 4:**
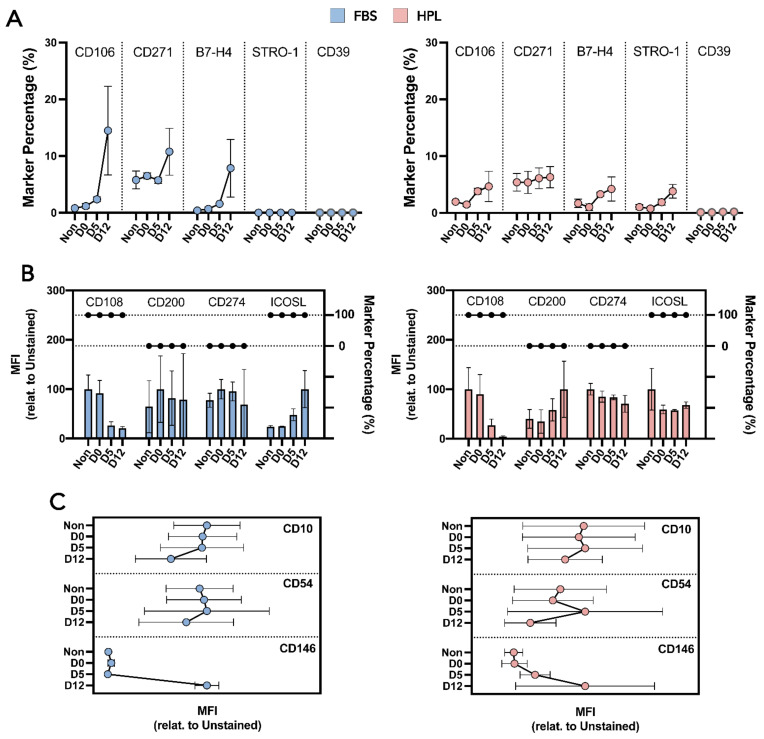
Characterization of MSC(AT) immunosuppression potential and clonogenic ability. (**A**) Subpopulation immunosuppressive and clonogenic marker expressions for MSC-FBS (**left**) and MSC-HPL (**right**). (**B**) Homogeneous immunosuppressive and clonogenic populations with marker percentage and median fluorescence intensity (MFI) levels for MSC-FBS (**left**) and MSC-HPL (**right**). The interconnected dots are the marker percentage and the bars are the MFI. (**C**) MFI analysis for motility (CD10), translocation (CD54), and hematopoietic support-related (CD146) markers for MSC-FBS (**left**) and MSC-HPL (**right**). Non—non-encapsulated; three MSC(AT) donors; mean ± SEM.

MSC-FBS and MSC-HPL immunophenotypes were extensively characterized via flow cytometry analysis to uncover whether released MSC-FBS and MSC-HPL maintained their identity and function. MSC identity markers (positive and negative) were tracked and did not change their expression throughout encapsulation (Figure 3B) [28,29]. Multiple MSC(AT) clonogenic and immunosuppression markers were also followed and showed different expression behavior (Figure 3C). Those that gave rise to positive subpopulations (e.g., B7-H4—immunosuppression and CD271—clonogenic), showed an increasing trend as encapsulation time grew for both MSC-FBS and MSC-HPL (Figure 4A). Median fluorescence intensity (MFI) analysis was done to detect variations in expression over time for markers where MSC(AT) displayed a homogeneous expression (Figure 4B). No significant differences in MFI were observed for these markers. Going further into MSC function, a small set of particular markers were also studied, namely motility-related CD10, trans-endothelium migration-related CD54, and hematopoietic support-related CD146 (Figure 4C). MFI tracking was able to discern an increase in CD146 expression as MSC(AT) reached D12 of encapsulation (Figure 4C). However, this MFI rise was not enough to give rise to a positive population or subpopulation as MSC-FBS and MSC-HPL maintained their negative expression (Figure 3C).

MSC(AT) preserved their identity during encapsulation as a homogeneous population for the set of markers analyzed. For MSC-FBS and MSC-HPL, numerous functional branches were explored and shown to be stable during storage at hypothermic temperatures.

### 3.4. Encapsulation Time Did Not Impact the Hematopoietic Support Capacity of MSC(AT)

**Figure 5:**
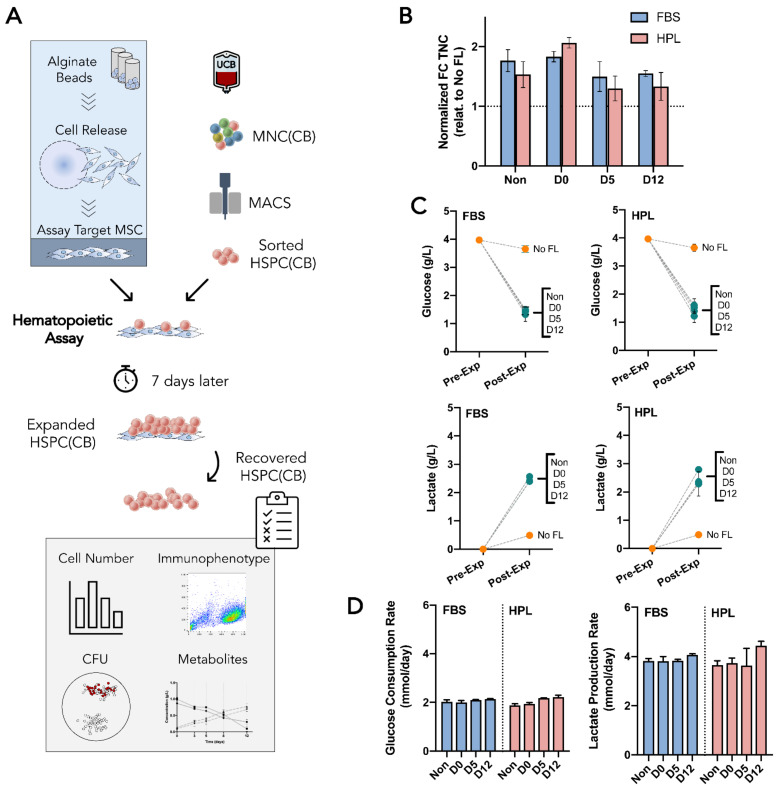
Hematopoietic support assay for MSC(AT) potency/function. (**A**) Experimental layout. Non-encapsulated and released MSC(AT) are replated as a feeder layer to investigate their hematopoietic support capacity. Umbilical cord blood-derived hematopoietic stem and progenitor cells (HSPC(CB)) were isolated via magnetic activated cell sorting (MACS) and seeded onto the MSC(AT) feeder layer. After 7 days in a co-culture setting, expanded HSPC(CB) were harvested and analyzed concerning their cell number, immunophenotype, metabolic activity, and differentiation potential using colony forming unit (CFU) assay. (**B**) Mean fold change (FC) in total nucleated cell (TNC) number after HSPC(CB) expansion normalized to the control condition (HSPC(CB) expanded without an MSC(AT) feeder layer (No FL). (**C**) Glucose (**top**) and lactate (**bottom**) concentration profiles for co-cultures of HSPC(CB) and MSC-FBS (**left**) and MSC-HPL (**right**). (**D**) Glucose consumption (**left**) and lactate production (**right**) rates during hematopoietic expansion. No FL—the control condition without an MSC(AT) feeder layer; Non—non-encapsulated; three MSC(AT) donors; mean ± SEM.

A hematopoietic support assay was proposed as a potency/functional assay for mesenchymal stromal cells (MSC) and was used to further evaluate the functionality of encapsulated cells (Figure 5A). Hematopoietic stem and progenitor cells (HSPC) from umbilical cord blood (UCB) (HSPC(CB)), known for expressing CD34, were sorted and co-cultured with a feeder layer (FL) of MSC(AT). In vitro expansion of HSPC(CB) within this co-culture system was evaluated with MSC(AT) as feeder layers (MSC FL) from each encapsulation timepoint and MSC expansion medium. Each HSPC(CB) expansion had an internal control where HSPC were cultured without an MSC FL.

Concerning the expansion fold change (FC) in total nucleated cells (TNC), no statistical differences were found between FL made by non-encapsulated MSC(AT) and encapsulated MSC(AT) (Figure 5B). Non-encapsulated MSC(AT) were able to support the expansion of HSPC(CB) up to a normalized 1.8-fold and 1.5-fold for MSC-FBS and MSC-HPL, respectively. This advantage in expanding HSPC with a FL co-culture was never lost, even though FL were prepared with MSC(AT) with increasingly longer encapsulation times. However, a slight decreasing trend was present, apparently causing the FL advantage to shorten (Figure 5B).

Cell metabolism during HSPC(CB) expansion was also followed to detect any possible changes in MSC behavior due to encapsulation. Here, due to the nature of a co-culture, both MSC and HSPC contributed to the metabolic dynamics observed. In both profiles (glucose and lactate), the co-culture led to more exhausted media due to its inherent higher cell number in culture than its control without FL (Figure 5C). Whether looking at glucose consumption or lactate production, co-culture of hematopoietic progenitors with MSC-FBS or MSC-HPL from the different encapsulation timepoints appeared to be very similar and did not seem to point to any metabolic changes. Quantification of metabolic rates confirmed that, metabolically, MSC(AT) FL established from previously encapsulated cells did not change their properties with the encapsulation process or encapsulation time (Figure 5D).

**Figure 6:**
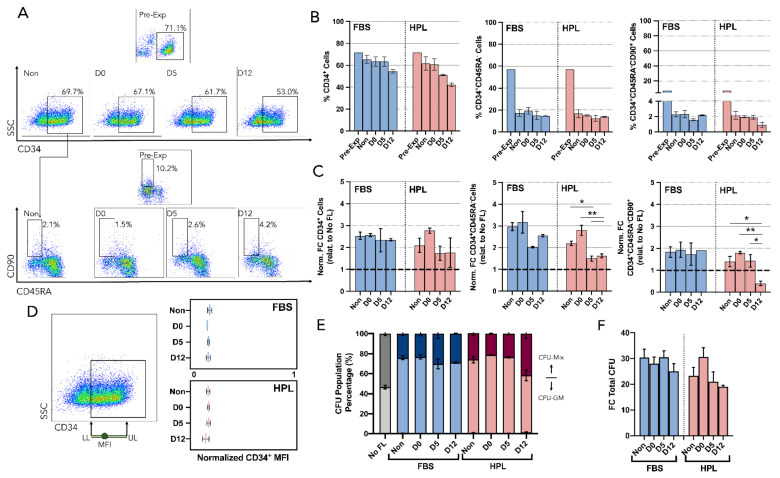
Immunophenotype and clonogenic potential (CFU) of hematopoietic stem/progenitor cells co-cultured with MSC(AT). (**A**) Representative dotplots showing the gating strategy used for the identification of different HSPC populations before expansion (Pre-Exp) and after expansion using released (D0, D5, and D12) or non-encapsulated (Non) MSC(AT) as feeder layers. Live HSPC were gated on forward scatter (FSC) versus side scatter (SSC) followed by the use of a viability dye. Then, CD34 expression was identified (**top**) and CD45RA and CD90 expression were also investigated to explore the remaining populations (**bottom**). (**B**) FC of normalized CD34^+^ (relative to the control No FL) (**left**), CD34^+^CD45RA^−^ (**center**), and CD34^+^CD45RA^−^CD90^+^ (**right**). (**C**) Percentage of CD34 expression (**left**), CD34^+^CD45RA^−^ (**center**), and CD34^+^CD45RA^−^CD90^+^ (**right**). (**D**) Quantification of CD34 loss after expansion. Mean fluorescence intensity (MFI) of CD34^+^ expression was quantified and normalized using the width of the positive CD34 population. (**E**) CFU population percentage. Neglectable burst-forming unit-erythroid (BFU-E) led mainly to two populations, including colony forming-unit granulocyte (CFU-GM) and colony forming-unit multilineage (CFU-Mix). (**F**) FC in total CFU number after HSPC expansion using FL from encapsulated and non-encapsulated MSC(AT) which were previously expanded in FBS or HPL supplemented medium. No FL—control condition without an MSC(AT) feeder layer; Non—non-encapsulated; SSC—Side scatter; LL—Lower limit; UL—Upper limit; three MSC(AT) donors; mean ± SEM, * *p* < 0.05, ** *p* < 0.01).

The impact of an MSC(AT) FL on the ex-vivo expansion of HSPC(CB) was further explored by identifying and quantifying different hematopoietic populations. Cell populations with ever increasing stemness were tracked (CD34^+^, CD34^+^CD45RA^−^, and CD34^+^CD45RA^−^CD90^+^) by immunophenotyping (Figure 6A). Feeder layers formed by released MSC(AT), either MSC-FBS or MSC-FBS, had comparable impacts on CD34 expression of expanded HSPC. Both types of FL caused decreasing trends as time of MSC encapsulation increased, and MSC-HPL FL, specifically, had an expression decline from close to 60% down to around 40% (Figure 6B). Interestingly, regarding the expansion levels (FC), CD34^+^ cells increased their numbers in a similar fashion between all conditions (encapsulation time and MSC expansion medium), namely around normalized 2.4-fold (Figure 6C). The progenitor population CD34^+^CD45RA^−^ had post-expansion percentages with no considerable differences concerning increased MSC encapsulation time. In contrast, expansion levels (FC) for CD34^+^CD45RA^−^ cells oscillated, with reduced levels for FL prepared with MSC(AT) encapsulated for longer periods. Concerning the more primitive HSPC population (CD34^+^CD45RA^−^CD90^+^), with a percentage before expansion of around 7%, no obvious differences in post-expansion percentages were noticed. D12 for MSC-HPL contributed toward a decreasing trend as it reached a positive percentage of 0.9% (Figure 6B). Overall, expansion FC followed suit; however, D12 for MSC-HPL became the only condition with a significant decrease under the normalization line (Figure 6C).

Median fluorescence intensity (MFI) of CD34-expressing cells at the end of each expansion was also followed to take advantage of the dynamics of CD34 expression where the loss of CD34 during the expansion is gradual and continuous (Figure 6D). No significant distinctions could be made between encapsulation conditions for both MSC-FBS and MSC-HPL.

The colony-forming unit (CFU) assay, which tested the myeloid differentiation potential of HSPC, was performed as part of the hematopoietic support assay. Cell culture without MSC FL typically originates an equal share of colony-forming unit granulocyte-macrophage (CFU-GM) and CFU-multilineage (CFU-Mix) for the expanded UCB cells, although the co-culture system increases the proportion of CFU-GM [27,30]. This difference was used to detect any loss of function by MSC(AT) during encapsulation. MSC-FBS were able to maintain this difference throughout the multiple encapsulation timepoints (Figure 6E). Similar to the results concerning the percentage of CD34^+^ and CD34^+^CD45RA^−^CD90^+^ cells, D12 for MSC-HPL also showed a slight increase in the proportion of CFU-Mix. Nevertheless, FC in the total CFU number of expanded HSPC showed that levels obtained by co-culturing them with encapsulated MSC(AT) were similar to co-culturing them with non-encapsulated MSC(AT) (Figure 6F).

**Figure 7:**
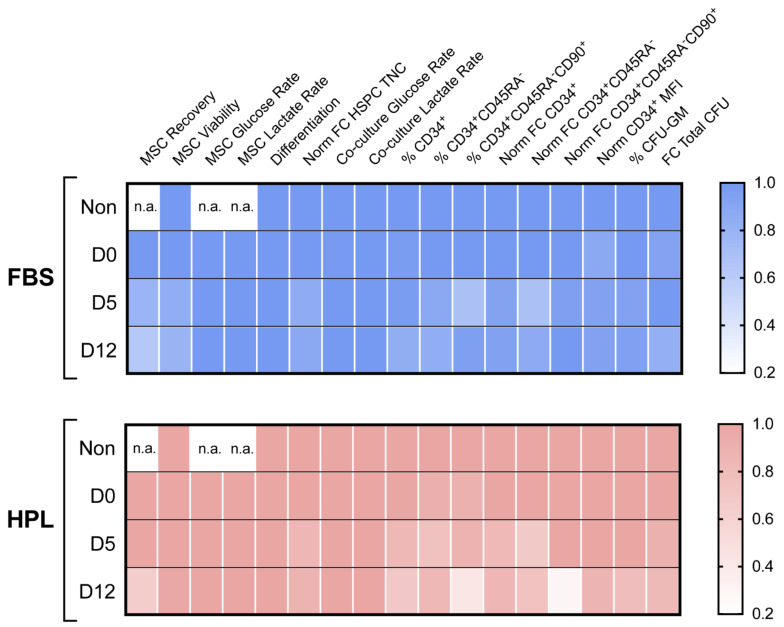
Heatmap score of MSC(AT) encapsulation. Key variables were put side-by-side to perform a comprehensive comparison between encapsulation timepoints for MSC-FBS (**top**) and MSC-HPL (**bottom**). Each variable was individually normalized using the value of non-encapsulated cells or D0 when non-encapsulated cells were not available. Differentiation was set to 1 for every timepoint as every differentiation was successful. Non—non-encapsulated; Glu—Glucose; Lact—Lactate; Norm—Normalized; FC—Fold Change; MFI—Median Fluorescence Intensity; n.a.—not applicable; three MSC(AT) donors; mean.

Considering every MSC-related variable studied, encapsulated MSC(AT) demonstrated mostly preservation of identity and functionality for the different encapsulation periods tested (Figure 7).

## 4. Discussion

Cell and gene therapies (CGT) are becoming a new reality for the treatment for multiple diseases ranging from cancer to auto-immune conditions [31]. With their approval gaining traction, several novel cell and cell-derived products may enter the market in the coming years [32]. This paradigm shift from traditional biopharmaceuticals to innovative CGT has unlocked more powerful therapeutic means to tackle diseases. The complexity involved in the manufacturing of these products has skyrocketed as we gain more specificity (e.g., CAR-T or CAR-NK targeting a particular tumor) or take advantage and augment existing cellular processes (e.g., mesenchymal stromal cells (MSC) for immunoregulation of auto-immune-derived wounds) [8]. The existence of technical challenges in the development of functioning production processes for CGT have led to threats against their commercial viability [8]. CGT risk reaching unrealistic prices upon reaching the market since the production cost has a direct influence on the price tag of the final approved therapy.

Tackling these challenges has made production cost reduction a key priority for the field. Determining the cost of goods (COGs) for the entire manufacturing pipeline is an efficient way to map production costs and uncover optimization opportunities. We knew this challenge exists for the entire production pipeline; therefore, in this study, we focused our ambition on impacting product storage and distribution. Although the amount of COGs studies has been limited and the uniqueness of each CGT manufacture makes it hard to generalize, therapy distribution has been said to account for up to 20% of the total manufacturing cost [10]. The costly burden of these two process steps—storage and distribution—can be partially justified by the use of cryopreservation (i.e., preservation at ultra-low temperatures below −130 °C) as the standard storage strategy for cellular therapeutics.

Cell cryopreservation can be considered a double-edged sword. On one hand, it makes it possible to stop biological time for large periods, thereby extending the lifespan of cellular products and maintaining their properties in an unaltered state. On the other hand, cell recovery from thawing has been a longstanding issue when handling cells. Post-thaw damage to cells is multifaceted, and its impact varies depending on the cell type. For MSC, cryopreservation has been thoroughly reviewed, and multiple studies have shown negative effects on viability, amount of apoptosis, attachment, and metabolism [33]. Furthermore, a growing concern in CGT is a cryo-stun effect after thawing. This effect appears to have special relevance for MSC since it is suspected to be a possible cause for the lack of success in their initial clinical trials [34]. Cryo-stun is a cell state where thawed cells display reduced potency or a dysfunctional phenotype. Differences between fresh and thawed MSC (e.g., cell growth, differentiation, and bioactivity) have been reported and reviewed [17]. Most of these adverse effects due to cryopreservation were shown to be temporary and were recovered after being cultured in vitro (i.e., cryorecovery or revitalization) [17]. Nevertheless, these strategies require additional costly and time-consuming handling before infusion and may be unrealistic at a large scale.

Cell encapsulation proposed herein completely circumvents the challenges of cryopreservation by using working temperatures that overlap the room temperature (RT) range (i.e., between 10 and 20 °C). This advantage can effectively eliminate an entire bioprocessing stage. BeadReady™ is currently available in the market and has an affordable price range; therefore, it contributes to making this technology readily available for potential CTG developers. In addition, a straightforward cell release step is available that involves a single solution exchange to facilitate full implementation by clinicians. Expensive and energy-consuming cryostorage equipment would no longer be necessary, nor would qualified labor with certification for handling cryogenic gases. The temperature-controlled containers with real-time temperature tracking would still be necessary to ensure the storage range is maintained; however, the distribution and supply chain model would change drastically since only a handful of companies dominate the market of cryo-temperature distribution (e.g., Marken or Cryoport) [35]. The ability to ship and distribute at warmer temperatures would open up a significant range of previously ineligible companies as potential partners, which may cause a very disruptive change in CGT logistics and supply chain.

Indeed, we showed adipose tissue-derived MSC (MSC(AT)) were able to survive encapsulated up to 12 days. By being able to do so with an adherent cell type (which normally requires surface adhesion and anchorage cues to survive), we unleash the potential of this method and this commercial kit for other non-suspension cells. We did observe a decline in the recovery percentage over time, but the values are similar to MSC thawing recovery by cryopreservation (around 75%) [36,37]. Importantly, cell viability was maintained over 70% throughout all timepoints and conditions. MSC(AT) were able to withstand warmer temperatures and showed few signs of deteriorating cellular health. Glucose consumption and lactate production were followed to determine the metabolic state of encapsulated MSC(AT) and their role in sustaining them over time since cryo-temperatures were not present to halt cell metabolism. MSC(AT) expanded in fetal bovine serum (FBS)-supplemented medium (MSC-FBS) and MSC(AT) expanded in human platelet lysate (HPL)-supplemented medium (MSC-HPL) appear to coalesce in their metabolic profiles during encapsulation, showing only slight distinctions in glucose consumption and lactate production behavior. Both specific glucose consumption and lactate production rates of encapsulated MSC(AT) determined in this study were consistently lower than previously reported values for non-encapsulated cells. Whether for umbilical cord tissue-derived MSC cultured in human serum-supplemented medium [38], bone marrow-derived MSC cultured in FBS-supplemented medium [25,39], MSC(AT) cultured in a commercially available xeno-free medium [40], or MSC(AT) cultured in HPL-supplemented medium in a bioreactor system [41], the reported specific metabolic rates are always, at least, one order of magnitude higher. Of note, encapsulated MSC(AT) were still subjected to a degree of hypothermic temperatures (i.e., between 10 and 20 °C, lower than the physiological 37 °C). Storage at such temperatures combined with the increased diffusion limitations present in alginate encapsulation may explain a slower metabolic state for encapsulated cells. A slower cell metabolism is typically associated with cell preservation which supports the use of alginate encapsulation for MSC storage and transportation. Interestingly, a degree of nutrient exhaustion was present at D12 that coincided with lower values of cell recovery. Unlike for MSC-FBS, where a downward trend was already in place, MSC-HPL cell recovery levels were stable until D12. Running out of available glucose may have been responsible for the observed loss of cell recovery, especially for MSC-HPL. Nutrient limitations should be considered, especially when defining the storage or transportation duration. Storage medium changes or higher initial glucose concentrations could be considered to potentially prevent undesired nutrient and metabolite levels.

The use of alginate as an encapsulation material has always shown promise for applications in cell therapy; however, so far, it has not been translated to a clinical scenario. Efforts are being made to create high-scale production strategies with good manufacturing practices (GMP) compliance [42], so alginate encapsulation may finally push through as a viable option for CGT. Some groups have explored hypothermic storage using alginate encapsulation. Human umbilical vein endothelial cells (HUVEC) were shown to maintain around 70% viability after 7 days of encapsulation [43]. After 3 days, 85% of encapsulated MSC(AT) were recovered and were able to reattach to a culture surface [23]. Encapsulated human limbus-derived MSC were able to sustain 5 days of hypothermic temperatures, with a recovery of close to 65% and a viability of 77% [44]. Of note, these encouraging results with limbus-derived MSC led to participation in an ongoing clinical trial (Identifier: CTRI/2021/07/035034). Our alginate encapsulation strategy led to similar or better values of recovery and viability for those specific timepoints. To our knowledge, this is the first study demonstrating the feasibility of having MSC(AT) encapsulated under hypothermic conditions up to 12 days.

We focused on tackling the translational challenges of alginate encapsulation and investigated the effect of alginate encapsulation on MSC(AT) with FBS supplementation, which is a standard for MSC culture, and HPL supplementation, which is a xeno-free alternative that is more amenable to clinical translation. Validation of HPL over FBS as a next-generation supplement to improve the clinical production of therapeutic MSC has been pursued by several groups and has been extensively reviewed [45]. In addition to eliminating the risk of potential immunogenicity and the transmission of zoonotic diseases associated with FBS, HPL was overwhelmingly shown to increase MSC proliferation while maintaining their immunophenotypic identity and differentiation potential [45]. However, HPL has also been associated with reduced MSC immunomodulation caused by an altered secretome and impaired inhibition of T- and NK-cell proliferation [46,47]. Additionally, the hematopoietic support capacity of MSC was demonstrated to be negatively affected by HPL supplementation as it was unable to retain certain hematopoietic stem and progenitor cell (HSPC) populations [30]. In our study, cells maintained in HPL-containing medium had slightly better encapsulation recovery and viability over time compared to FBS-based medium and comparable performance concerning the remaining assays. Cell encapsulation for MSC transportation and storage has been proven to be compatible with an animal component-free culture supplement, contributing toward a fully GMP-compliant MSC manufacturing process and promoting its use in a clinical setting.

We sought to confirm whether MSC(AT) identity and function were preserved throughout their encapsulation when we evaluated this commercially available alginate-based encapsulation kit. The need for reliable MSC functional or potency assays is a long-standing issue in the field [48,49]. Depending on the therapeutic goal, MSC therapies might require different readouts of potency. Taking this into consideration, the development of a set of assays that encompass most of the therapeutic value of MSC (i.e., immunomodulation, tissue regeneration, homing, etc.) instead of relying on a single one may be the future of MSC manufacturing. Such a matrix of assays has been proposed and is still being refined [50,51]. We proposed a novel hematopoietic support potency assay in order to contribute to the efforts of establishing a potency matrix platform for clinical-grade MSC. MSC-HPSC co-culture is considered one of the main expansion platforms for clinical HSPC [52] and is currently in the clinical trial pipeline [53]. MSC also have a supportive role in hematopoietic cell co-transplants as they assist with the engraftment of transplanted HSPC and reduce conditioning-related bone marrow inflammation [54]. In this co-culture system, MSC feeder layers (FL) were shown to confer HSPC an advantage during their expansion compared with systems that only use exogenous cytokines [27,55]. This hematopoietic support ability of MSC can be quantified and used as an indicator for MSC potency or function. Our group has an extensive background with this co-culture expansion system and has contributed toward its translation potential [27,30,56,57,58,59,60,61,62]. Our proposed hematopoietic support assay has the advantage of multiple quantifiable readouts (e.g., HSPC expansion fold change, metabolite quantification, HSPC immunophenotype, and percentages of colony-forming unit [CFU] populations) and an internal control (i.e., HSPC expansion only with exogenous cytokines). In our case, considering all the assay readouts, MSC(AT) from both expansion media demonstrated that their encapsulation did not have an impact on their functional properties related to hematopoietic support. With this precedent, we consider that the MSC potency matrix could only benefit from the inclusion of a hematopoietic support assay in its ranks.

We lay the ground for more ambitious goals by unblocking the access of product storage and transportation to alginate encapsulation. The development of an entire cell therapy manufacturing process using encapsulated cells may now be possible. This all-in-one strategy would allow MSC or other cell types to remain encapsulated from an initial manipulation step to the final infusion into the patient. Both cell manipulation and the clinical administration of encapsulated cells have been widely explored for a great variety of applications. However, a bridge between these process units has been lacking. Concerning cell manipulation, 3D-expansion of MSC(AT) in alginate core-shell capsules has been shown to be possible as it obtained a modest 2.5-fold increase after 4 days [63]. Paracrine activity of MSC was also proven to be compatible with alginate encapsulation, with angiogenic and chemotactic factors measured from encapsulated MSC [64]. Genetic manipulation of MSC in alginate beads to direct their phenotype to a more osteogenic or chondrogenic state as a cartilage or osteochondral tissue engineering approach was likewise successfully demonstrated [65]. These examples validate the compatibility of alginate encapsulation and the different therapeutic avenues of MSC. Alginate has a substantial clinical safety record concerning administration into patients [66]. Alginate-based islet and β-cell encapsulation have had success in enabling in vivo glycemic control in Type 1 diabetes models, with several novel encapsulation systems currently in clinical trials [12,67]. Besides being safe and biocompatible, alginate encapsulation can also potentially address some of the challenges of MSC translation, particularly a lack of cell retention. In vivo presence of encapsulated immunomodulating MSC was substantially increased after intravenous injection in mice [68]. In a myocardial infarction mouse model, encapsulated MSC were detected in higher numbers after 7 days and reduced scar formation and demonstrated a superior angiogenesis when compared to free MSC [69]. Overall, our study has significantly contributed toward the feasibility of bridging cell manipulation and infusion using alginate encapsulation, which makes it possible for encapsulated cells to be temporarily stored and transported at convenient temperatures from their manufacturing and manipulation sites to their therapeutic administration.

## 5. Conclusions

Adipose tissue-derived mesenchymal stromal cells (MSC(AT)) were successfully demonstrated to be compatible with cell encapsulation at hypothermic temperatures (10–20 °C). Maintenance of identity markers was ensured, while MSC(AT) multi-layered functionality was proven to be maintained throughout encapsulation. Differentiation, expression of immunomodulatory molecules, and hematopoietic support abilities were all individually confirmed. Translation of clinical-grade MSC(AT) to this novel product storage and transportation model appears to be within reach. Xeno-free processing of MSC(AT) was directly compared with traditional fetal bovine serum (FBS)-based handling. Human platelet lysate (HPL) supplementation, which by itself improves MSC proliferation in vitro, did not negatively impact cell behavior during our encapsulation study, which makes it a relevant candidate for FBS substitution for good manufacturing practice (GMP)-compliant MSC logistics and supply chain. As a model, cell encapsulation will potentially have a disruptive impact on cell and gene therapies (CGT) as it does not require cryo-range temperatures (below −130 °C). Alginate encapsulation also has the potential to be complementary to cryopreservation as it is appropriate for both allogeneic and autologous scenarios. Optimization opportunities exist (e.g., improving initial encapsulation efficiency) and should be pursued to further enhance encapsulation potential.

## Data Availability

Not applicable.
